# Missing Values in Longitudinal Proteome Dynamics Studies: Making a Case for Data Multiple Imputation

**DOI:** 10.1021/acs.jproteome.4c00263

**Published:** 2024-08-27

**Authors:** Yu Yan, Baradwaj Simha Sankar, Bilal Mirza, Dominic C. M. Ng, Alexander R. Pelletier, Sarah D. Huang, Wei Wang, Karol Watson, Ding Wang, Peipei Ping

**Affiliations:** † Departments of Physiology and Medicine, University of California, Los Angeles (UCLA) School of Medicine, Los Angeles, California 90095, United States; ‡ NHLBI Integrated Cardiovascular Data Science Training Program, UCLA, Los Angeles, California 90095, United States; § NIH BRIDGE2AI Center & NHLBI Integrated Cardiovascular Data Science Training Program, UCLA, Suite 1-609, MRL Building, 675 Charles E. Young Drive South, Los Angeles, California 90095, United States; ∥ Department of Computer Science and Scalable Analytics Institute, UCLA School of Engineering, Los Angeles, California 90095, United States

**Keywords:** data imputation, multiple imputation, protein turnover rate, longitudinal data

## Abstract

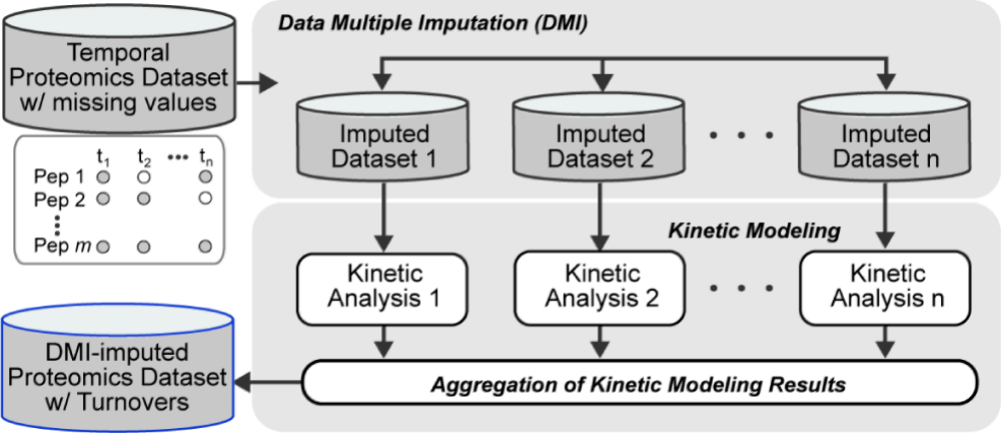

Temporal proteomics data sets are often confounded by the challenges of missing values. These missing data points, in a time-series context, can lead to fluctuations in measurements or the omission of critical events, thus hindering the ability to fully comprehend the underlying biomedical processes. We introduce a Data Multiple Imputation (DMI) pipeline designed to address this challenge in temporal data set turnover rate quantifications, enabling robust downstream analysis to gain novel discoveries. To demonstrate its utility and generalizability, we applied this pipeline to two use cases: a murine cardiac temporal proteomics data set and a human plasma temporal proteomics data set, both aimed at examining protein turnover rates. This DMI pipeline significantly enhanced the detection of protein turnover rate in both data sets, and furthermore, the imputed data sets captured new representation of proteins, leading to an augmented view of biological pathways, protein complex dynamics, as well as biomarker–disease associations. Importantly, DMI exhibited superior performance in benchmark data sets compared to single imputation methods (DSI). In summary, we have demonstrated that this DMI pipeline is effective at overcoming challenges introduced by missing values in temporal proteome dynamics studies.

## Introduction

Missing values, absence of observations for one or more variables in the data set, is a common challenge across a wide range of biomedical data sets,^1−4^ including proteomics data sets.^5,6^ Missing values can adversely impact data quality, subsequent downstream analysis and/or modeling, resulting in biased outcomes, and incomplete conclusions.^7^ Overcoming missing data points is essential for rendering a data set to be “AI-ready”, which refers to the data operations performed to meet the requirements of AI models.^8^ To appropriately address missing values, it is necessary to explore the factors contributing to them, including the conditions under which data sets were collected (e.g., experimental equipment^2,9,10^). In particular, missing values in temporal data sets, i.e., data sets with repeated measurements at multiple time points are further complicated by (1) the continuity of time series data, which might be hampered due to the proportion of missing values; and (2) any intrinsic temporal patterns, which are yet to be revealed. Ostensibly, addressing these complexities in temporal data sets requires context specific solutions.

The advancement of proteomics technologies, e.g., tandem mass spectrometry (MS),^11,12^ has rendered proteome-wide examinations and measurements of protein dynamics feasible with unprecedented detail.^13,14^ Despite significant advancements in technology, MS-based proteomics often grapples with the issue of missing values. Missing values in proteomics can arise from a variety of factors, including peptide abundances that fall below the detection limit, error from laboratory preparation or instrumentation and/or data processing.^15,16^ When/if a significant portion of peptide data are absent, the subsequent quantification of protein expressions as well as measurements of protein turnover rates will be affected.^17^ Accordingly, missing turnover rates and inaccurate turnover rate estimation may occur with incomplete time series when the number of peptides quantified across time points is insufficient for model fitting. This issue introduces biases in subsequent analyses, thus hindering biological discovery and understanding.^5,6,18^ Seminal works have been implemented to tackle these issues in protein expression data,^2,5,6,19−23^ whereas effective approaches specifically addressing missing values in the context of temporal dynamics profiling are lacking. Accurate estimation of protein turnover rate is contingent upon a complete time-series data set and is more vulnerable to missing values.^24,25^

Generally, data imputation methods can be classified into single- and multiple imputation approaches. Most imputation methods applied in proteomics are single imputation techniques, where each missing value is filled by one imputed value.^2,5,6^ Although single-imputation approaches are widely adopted, estimates from single imputation are treated as observed values, making them indistinguishable in downstream analyses. Single imputation falls short of capturing the uncertainty associated with missing values, often resulting in unrealistically narrow standard errors.^26^ In contrast, Data Multiple Imputation (DMI) methods address these challenges and have been applied on nontemporal proteomics data set.^23^ DMI generates multiple imputations for each missing value, allowing for the aggregation of these imputations to derive a final imputed value. DMI considers variability across imputed data sets, thereby reflecting the inherent uncertainty in missing values, an aspect not addressed by single imputation methods. Moreover, DMI methods can be seamlessly integrated with downstream analysis. For example, for protein turnover rate estimation, imputed values will not be distinguished from observed values, leading to potential overreliance on the imputed data and skewing estimates. DMI imputes multiple values for the same missing values via sampling from posterior distributions of the parameters, better capturing the uncertainty during the process. Then the protein turnover rate can be inferred from each imputed data set individually and then pooled to derive final parameter estimates, therefore better addressing the potential variation from the imputation. In addition, DMI utilizes time series from other peptides to capture the potential temporal dependency via Fully Conditional Specification (FCS).^27^ Therefore, the DMI integrated workflow takes into consideration temporal dependencies, uncertainties at single time point, as well as time series levels to address the multilevel challenges introduced by missing data in temporal proteomics studies.

**Figure 1:**
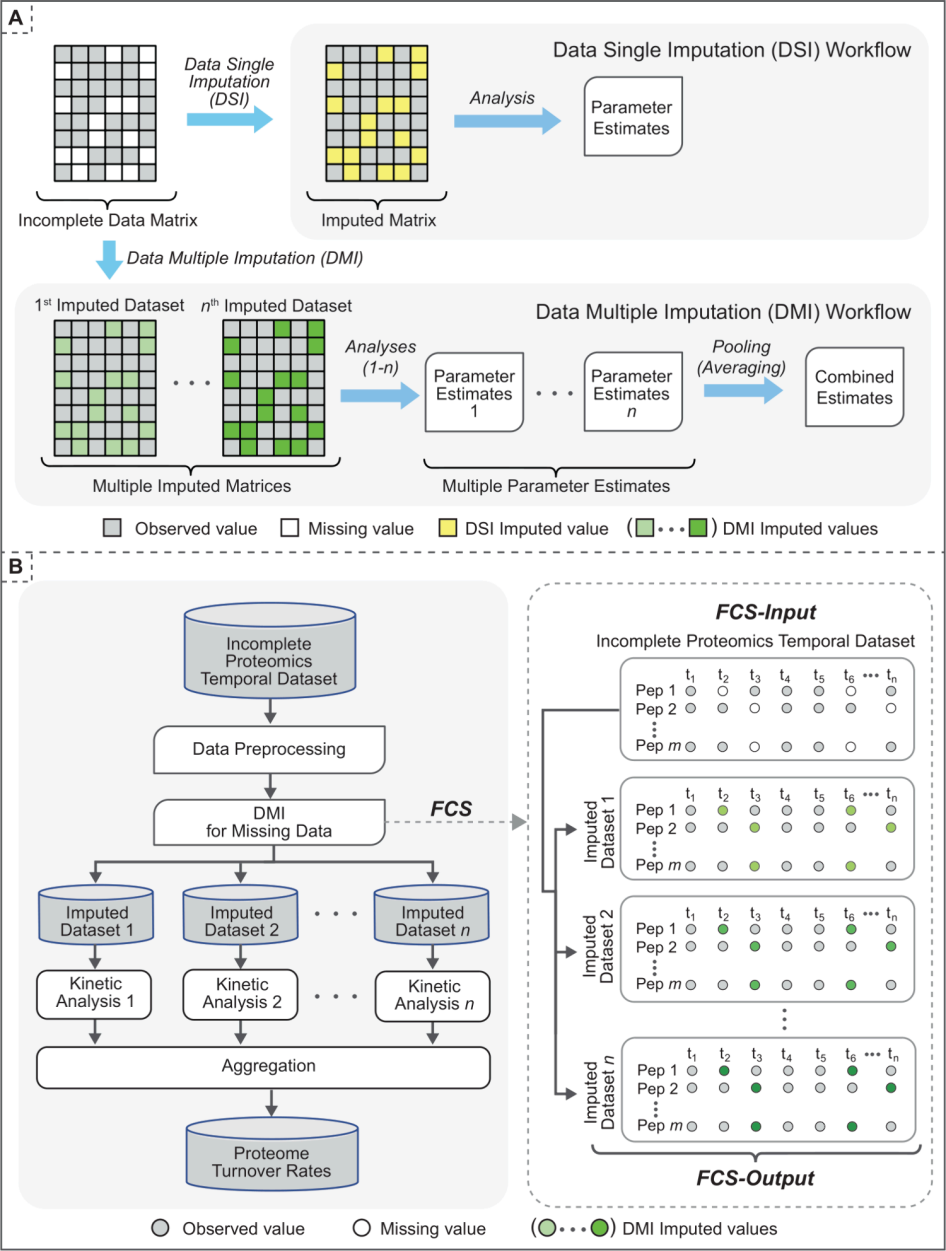
Data imputation workflows. (A) Data Single Imputation (DSI) and Data Multiple Imputation (DMI). In the DSI approach, each missing value (white cell) in the incomplete data matrix is replaced with a single estimate (yellow cells). Imputed values are treated as observed values in the imputed data matrix for downstream analysis. In the DMI approach, multiple values are imputed for each missing value in the incomplete data matrix. Consequently, there are multiple imputed data matrices with the same observed values but different imputed values (green cells). Analysis of each imputed data matrix is performed separately, and the final estimates are obtained by pooling the results from multiple analyses. (B) DMI for missing values in Proteome turnover Data set. The DMI pipeline computed protein turnovers from an incomplete temporal data set (peptide isotope intensities). As data preprocessing, we included peptides detected at ≥2 time points. The missing values were imputed using Fully Conditional Specification (FCS). DMI generated 10 imputed data sets in which peptide isotope intensity values are imputed at each of the time points (*t*_1_ – *t*_*n*_) when/if the data was missing. Each data set has the same observed values but slightly different imputed values. Kinetic analysis^25^ was performed on each imputed data set independently, and the protein turnover rates were obtained by averaging the results of multiple analyses.

We have developed a DMI pipeline to effectively address missing values in estimating protein turnover rates from time series proteomics data. Our workflow (Figure 1B) showcased its effectiveness and generalizability on a cardiac temporal proteomics data set from mice and a temporal plasma proteomics data set from humans.

## Experimental Procedure

### Data Sets

#### Murine Data Set

A temporal proteomics data set characterizing large-scale cardiac protein turnovers across multiple mouse strains.^28^ To summarize, this study is divided into two groups: Isoproterenol (ISO) treated mice and Controlled (Ctrl) mice were metabolically labeled with deuterium water. Within each group, six mouse strains were used: A/J, BALB/cJ, C57BL/6J, CE/J, DBA/2J, and FVB/NJ. From each experimental group, two mice were euthanized on each day: 0, 1, 3, 5, 7, 10, and 14 to collect heart and plasma samples. In the cardiac hypertrophy groups, surgical implanted subcutaneous micro-osmotic pumps (Alzet) were calibrated to deliver 15 mg·kg^–1^·d^–1^ of isoproterenol over 14 days.

#### Human Data Set

A human temporal proteomics data set that performed high-throughput quantification of protein turnover in ten human subjects.^29^ This proteomics data was acquired from healthy human plasma samples collected at ten defined intervals: days 0, 1, 2, 4, 5, 8, 9, 10, 12, and 14.

The peptide samples from both data sets were analyzed by liquid chromatography-tandem mass spectrometry (LC-MS/MS) to discern peptide abundance, isotope incorporation, and sequences. Protein turnover kinetics and estimated fitting errors were analyzed through “Proturn”.^30^ Additional details of the data set can be found in previous publications.^24,25^

### Construction of the Data Multiple Imputation (DMI) Pipeline

We incorporated FCS in our pipeline using the R package “MICE”.^31^ We formatted the data from both data sets as a proteome-wide time series of A0 (the fraction of the zeroth isotopomer of a peptide isotope envelope, which is used to estimate the protein turnover rate). For the murine data set, this was done for each mouse strain in each condition (ISO/CTRL), and for the human data set, for each healthy subject. Missing A0 values at any given time point were imputed based on the remaining time points.^26^ If multiple A0s from different peptides in the same proteins exist, the median of the A0s was used. The imputation was performed on the peptides that have at least two observed time points; this is not to be confused with the requirement of four time points to perform the turnover rate estimation. We used FCS to reproduce the correlations over time and set the number of imputed data sets, *m*, to 10. Subsequently, we performed half-life computation with “Proturn” on the 10 resulting data sets separately, with identical settings. For any given protein, the final turnover rate constant *k* is the average rate constant estimated from 10 runs of half-life analyses. This process is repeated for each of the 12 samples, i.e., 6 samples under both ISO-treated and CTRL conditions, in the murine data set and for each of the 10 healthy subjects in the human data set. Compared to previous work, this pipeline is flexible to accommodate other types of DMI techniques and larger *m*, and provides a platform for comparing different approaches for missing data.

### “Proturn” for Computing Protein Turnover Rates

“Proturn” was used to calculate protein turnover kinetics and estimated fitting errors as previously described.^30,32^ “Proturn” automatically retrieved identified peptides that were uniquely assigned to proteins for the area integration. The “Proturn” parameters were set as follows: area-under-curve integration width: 60 ppm, extracted ion chromatogram smoothing: Savitzky–Golay filter over 7 data points. To further control against peptide false positive identifications, only peptides that were explicitly identified (1% FDR) and integrated in ≥4 time points were accepted for the calculation of protein abundance and turnover.

### Evaluation Framework for Missing Data Imputation

To simulate missing data scenarios, we first retrieved peptides from the murine cardiac temporal proteomics data set that contained a complete time series in A0 with no missing values, such that we can ensure that the turnover rate is estimated without missing values and can serve as a ground truth for evaluating the imputation methods. To simulate the different levels of missingness, we create five masked data sets where 1 up to 5 time points out of the complete 7 time points were randomly masked. On each of these masked data sets, we applied three imputation methods: (1) DMI; (2) Single imputation with mean; and (3) Single imputation with k-nearest neighbor (KNN) using 30 neighbors. Each masked data set that underwent the Data Single Imputation (DSI) workflow produced one imputed data set. Each masked data set that underwent the DMI workflow produced 10 imputed data sets for each of the masked configurations. Subsequently, we conducted kinetic analysis to quantify the turnover rates on each masked data set for each imputation method independently. The accuracy of the imputation methods was quantified using the normalized root-mean-square error (NRMSE)^22^ comparing the actual values versus the imputed values for A0 and turnover rates.

### Impact of DMI on Biomedical Insights

#### Summary of Number of Samples Available for Turnover Calculation with a Barplot

For each time series of a specific protein from different experimental conditions (6 strains × 2 treatments = 12 conditions), the number of nonmissing data points were counted (ranging from 0 to 7) by picking the peptide with the least missing values in the time series. The counts from different experimental conditions for the same protein are then aggregated to yield the total number of observations and the number of missing observations imputed for that protein. Proteins are sorted by the number of observations in the barplot. The barplot showing the numbers of proteins recovered by DMI under different conditions follows the same procedure.

#### Protein Expression Comparison on Proteins Quantifiable with or without DMI

Violin plots compare the abundance value (normalized spectral abundance factor, NSAF) and turnover rates between proteins only quantifiable by DMI and those quantifiable without DMI. The area of each violin is adjusted to reflect the number of proteins. A two-sample two-sided Wilcoxon test is performed, and the p-value is shown in the figure. The Wilcoxon test is performed in R using wilcox.test.

#### Reactome Pathway Enrichment Analysis

Reactome database was used to analyze the biological processes associated with the identified proteins, including those recovered through imputation methods.^33^ We performed Reactome Pathway enrichment analysis with the following settings: *Mus musculus* genes as the reference list; biological process complete as the annotation data set; Fisher’s Exact test and calculate FDR. The analysis was specifically designed to pinpoint biological processes that are significantly enriched in our data set of proteins, with an emphasis on contrasting those proteins identified through DMI with those not subjected to DMI. Biological processes that are only enriched in the protein list subjected to DMI are shown.

#### Protein Complex Stability Analysis

Protein complex information was retrieved from Complex Portal.^34^ We selected complexes for which all protein interactors in the complex were represented in the proteomics data set and focused on heterocomplexes, i.e., complexes with multiple protein interactors. Stability is calculated as the standard deviation of the average protein turnover rates within the protein complex. To compare against proteins sampled from the proteome, we account for the number of proteins in the complex by sampling from the proteome with the empirical frequency of the number of proteins in complexes. A Wilcoxon Test was performed to calculate the p-values. We also analyzed the dynamics of individual protein complexes across the experimental groups. Using one-way Analysis of Variance (ANOVA), we examined differences in the mean turnover rates of protein interactors in four complexes.

#### Biomarker Analysis on Human Temporal Proteomics Data set

MarkerDB is a professionally curated database of preclinical biomarkers.^35^ From this database, we identified 137 unique protein biomarkers and retrieved their UniProt IDs using UniProt KB API.^36^ We identified the intersection of these biomarkers and proteome quantified with and without imputation in the human temporal proteomics data set. We then queried MarkerDB to map the biomarker lists of each human subject to their disease associations in order to identify new or corroborated disease associations revealed by the additional imputed proteins.

## Results and Discussion

### The DMI Pipeline to Recover Temporal Proteomics Data with Flexibility

We developed a DMI pipeline capable of imputing missing values in temporal proteomics data, rendering greater coverage of protein turnover rates. Our workflow (Figure 1B) first preprocesses the temporal proteomics data set to fit the format required by DMI. DMI is then performed to impute missing values for m rounds, where m is predefined. The resulting m imputed data sets allow quantification of protein turnover rates for all identified proteins, a task that would have been challenging, and sometimes infeasible, with incomplete data sets. Kinetic analyses are performed on these data sets separately, leading to M estimates of protein turnover rates. Finally, all estimates are pooled to generate the final turnover rates, proteome wide.

### The DMI Pipeline Enhances the Final Determination of Protein Turnover Rates

**Figure 2:**
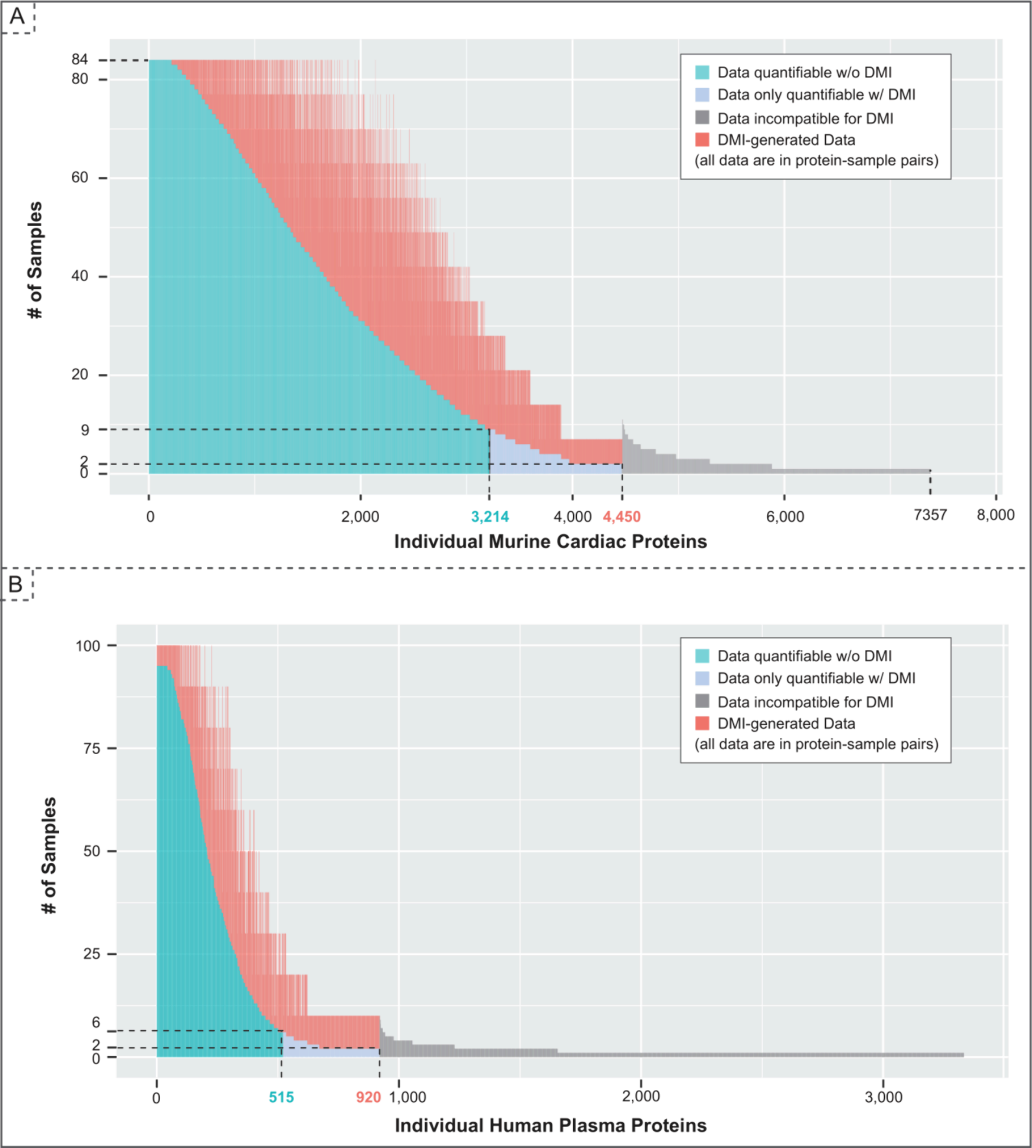
DMI improves coverage of the proteome turnover rates. Supporting evidence from two independent data sets are presented here. (A) The mouse data set contains 84 samples (6 strains × 2 treatments × 7 time points). The individual proteins are represented in the *x*-axis in decreasing order of samples, where their turnover rates were quantifiable without (blue) DMI and with DMI (red). Without DMI, the turnover rate of 3,214 proteins (in dark blue) were quantified. With DMI, the turnover rates of 1,236 (38%) additional individual proteins were quantified (in light blue), capturing a total of 4,450 protein turnover rates. Only a small fraction of samples (in gray, 2,907 proteins) did not satisfy our minimum requirement for imputation. (B) The human plasma data set consists of 100 samples (10 subjects × 10 time points). Similarly, without DMI, the turnover rates of 515 proteins (in dark blue) were quantified. With DMI, the turnover rate of 405 (78%) additional individual proteins were quantified (in light blue), capturing a total of 920 protein turnover rates.

Our DMI pipeline is able to fully utilize the information that can be extracted about proteome dynamics from the temporal proteomics data sets. In the previous analysis, peptides identified at least 4 times were selected to control false discovery rate of protein turnover quantification.^28,29^ The requirement for a minimal number of time points is to ensure adequate information for accurate turnover rate estimation. Our DMI pipeline captures a more complete proteome-wide turnover rate in both data sets. Thus, proteins that were previously quantifiable (>4 time points) but not present in the full time points also benefit from inclusion of DMI-imputed data for more accurate kinetic analysis. A detailed number of imputed samples and original samples for both data sets are shown in Figure 2.

We evaluated the performance of DMI on imputing missing values in comparison to single imputation methods (DSI). We developed an approach to introduce missing values by masking experimentally observed values for peptides’ with a complete time-series. To examine the temporal aspects of the imputation, we evaluated how well each imputation method can recover masked values and subsequently estimate turnover rates from the imputed time series. Across various levels of missingness, DMI consistently outperformed k-nearest neighbor (KNN) imputation and mean imputation in accurately imputing experimentally observed A0 values and turnover rates as measured by NRMSE (please see Supporting Information, Figure S1).

### The DMI Pipeline Ensures a Comprehensive View of Protein Turnover Rates

**Figure 3:**
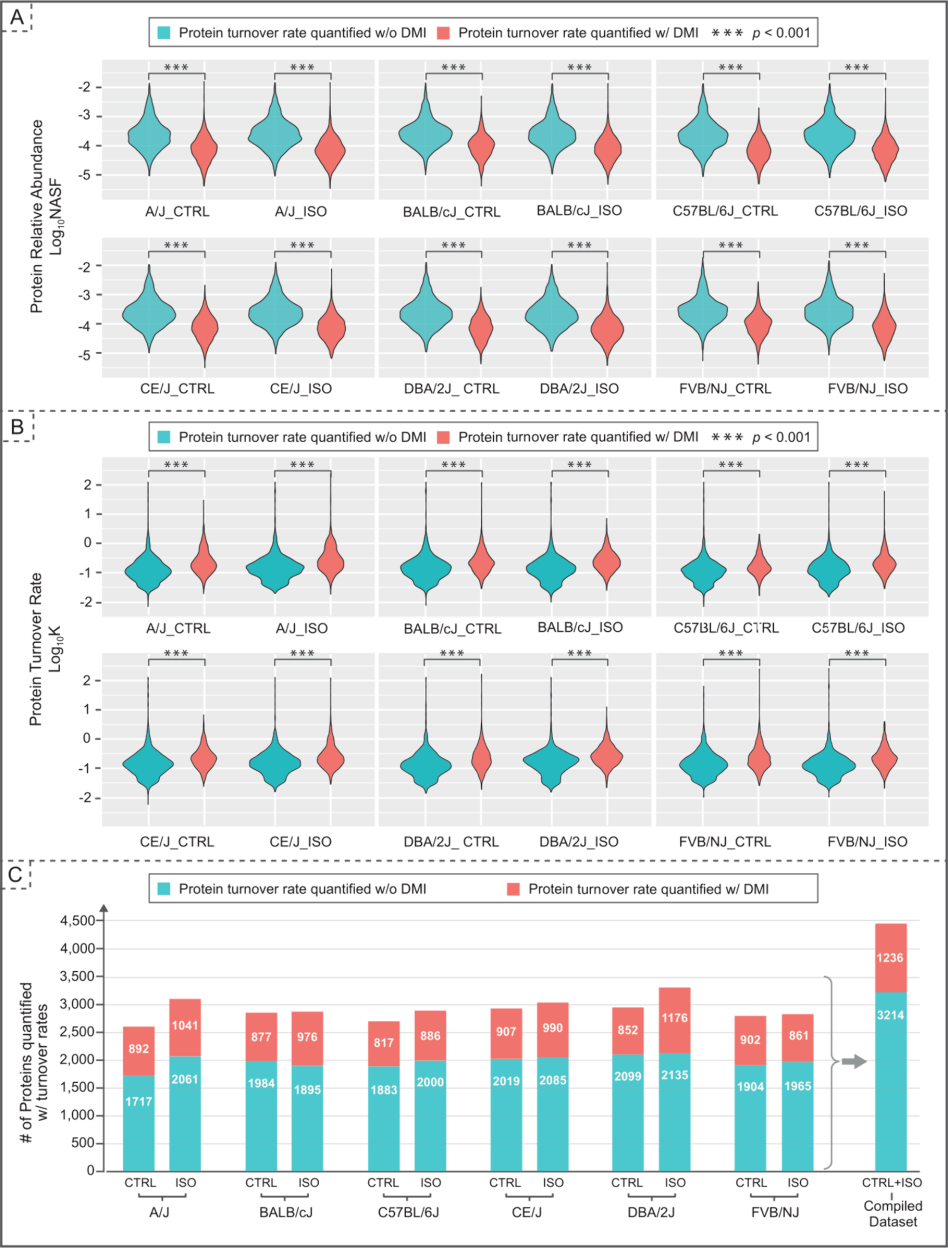
Impact of DMI on protein expression and turnover rate. (A) Violin plot shows the protein relative abundance of those with DMI (orange) and those without (blue), indicating that DMI has a more pronounced impact on proteins of lower abundance. (B) Violin plot shows the protein turnover rate computed from the data set with or without DMI, illustrating the DMI has a bigger influence on proteins with faster turnover rates. Statistical significance between groups in both violin plot is determined using the Wilcoxon test (***p-value <0.001). (C) Bar chart compares the quantifiable protein turnover rates with and without data imputation across six mouse strains. Data imputation leads to a 40–50% increase (orange) in the quantifiable turnover rates in each strain.

A detailed number of proteins quantifiable after imputation in each mouse strain under two conditions is shown in Figure 3C. Around 50% improvement of coverage is shown in all strains under both conditions. With the improved coverage, we have a more comprehensive view of the proteome dynamics landscape during cardiac hypertrophy pathogenesis.

As previously demonstrated with proteomics data, missing values are correlated with low abundances of the protein, i.e., proteins with low abundance were prone to contain missing values.^9^ We investigated whether low abundance also correlates with the missing values in the protein turnover rate. We further explored this relationship in the context of protein turnover rates. Specifically, we compared the abundance levels and turnover rates of proteins that can be quantified without DMI to those that are only quantifiable with DMI (Figure 3A and 3B). A significant difference in both the abundance and turnover rate between these two groups in all strains and two treatments suggests that the proteins with lower abundance and higher turnover are prone to be missing in the turnover rate calculation. Thus, DMI enables proteins with lower expression to be captured, ensuring a more comprehensive view of proteome wide protein dynamics (Figure 3B).

### The DMI Pipeline Captures a Broad Representation of Biological Processes

To investigate how an imputed data set can better capture the comprehensive biological processes of the proteome, we performed the Reactome Pathway enrichment analysis on both the protein sets before and after imputation to determine the potential loss of biological processes if no imputation is performed. There were 199 and 238 biological processes enriched from the proteins recovered with imputation in health and disease, respectively (Figure S2). In the healthy group, biological processes related to localization, autophagy, splicing and so on are enriched. In the disease group, biological processes related to transportation, splicing, and autophagy are enriched. While the recovered biological processes in the two groups were not the same, they share common pathways in terms of high-level processes such as splicing, localization and autophagy.

### The DMI Pipeline Reveals a Dynamic Landscape on Protein Complexes

The turnover rate of individual proteins within protein complexes offers insights into their stability, regulatory mechanisms, and functional lifespans, enhancing our understanding of cellular biology.^14,24,37^ We investigated the turnover rate landscape of multiple heterocomplex interactors, revealing the dynamic view of protein complexes.

**Figure 4:**
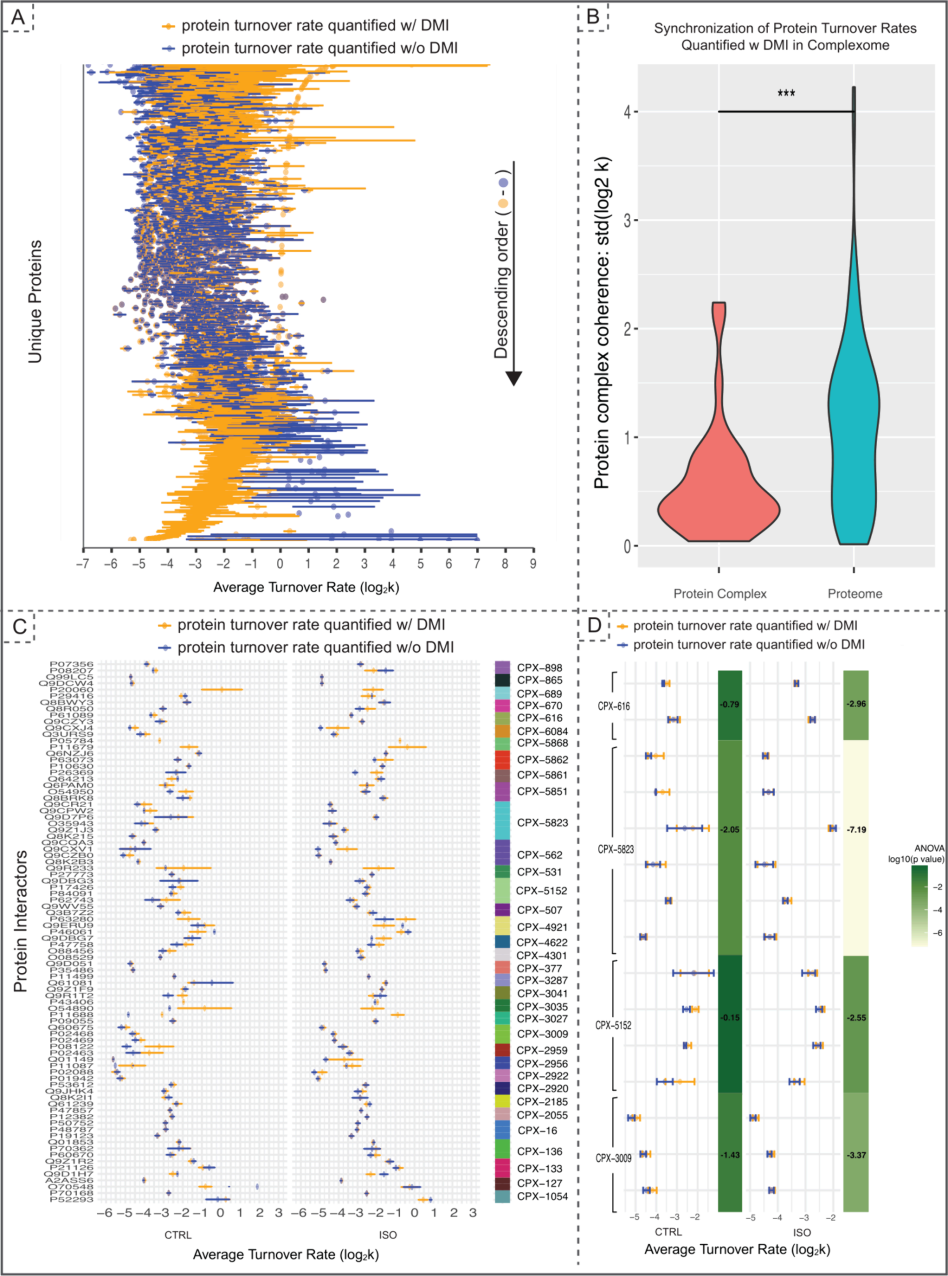
Impact of DMI on protein complex dynamics. (A) Scatter plot of proteome turnover rates from top to bottom based on the absolute impact of DMI on turnover rate estimations: enhancement (on the top), agreement (in the middle), reduction (in the lower part), or the assignment of an imputed value, previously unquantifiable in the absence of DMI. Each row represents a protein, and the rows are organized in a descending order of the difference between protein turnover rates estimated after and before imputation. Error bars represent standard error mean (SEM); they are 0 if *n* < 2. (B) A violin plot shows a pronounced synchronization of turnover rates among proteins within complexes, as evidenced by the standard deviation of the turnover rates quantified post-DMI compared to the broader proteome. “***” indicates a *p*-value <0.001. (C) A scatter plot of protein turnover rate within individual complexes, showing the impact of DMI on assessing the dynamic behavior of proteins within the same complex. A color bar indicates the protein complex the protein interactors belong to. Detailed examples are given in panel D. (D) A zoom in view of four protein complexes selected from panel C: UBC13-UEV1A ubiquitin-conjugating enzyme E2 complex; Mitochondrial NIAUFX iron–sulfur cluster assembly complex; AP-2 Adaptor complex, alpha1 variant; Laminin-211 complex, where DMI provides insight into the synchronized protein turnover behavior in CTRL which was disrupted in ISO.

We first explored the impact of DMI on proteome-wide turnover rates, revealing that DMI elucidates a detailed proteome turnover landscape (Figure 4A). While the majority of proteins show relatively consistent turnover rates before and after DMI, we observed increases and decreases of turnover rates as a result of increased time points imputed by DMI. The proteins that have lower turnover rates after imputation seem to have a large discrepancy before and after DMI. This discrepancy likely arises because these protein turnover rates, when quantified without DMI, are challenging to measure due to the high proportion of missing values that lead to fewer data points and greater variation across replicates. Subsequently, we investigated the turnover rates of proteins within heterocomplexes, characterized by the Complex Portal database.^34^ We defined a metric, the standard deviation of turnover rates, as a measure of the synchronization of turnovers within protein complexes. A lower standard deviation signifies a more coordinated complex, characterized by similar level protein turnover rates. Our analysis demonstrated that the synchronization of protein complexes was significantly greater than that observed for proteins sampled from the proteome, suggesting a coordinated regulation of turnover within the complexes (Figure 4B).

The turnover landscape offered by DMI allowed for an understanding of how individual complex dynamics may be coordinated across experimental groups (Figure 4C). Importantly, the ability to assess the dynamics of all protein interactors in certain heterocomplexes is only made possible by DMI (e.g., CPX-5868, 4921, 3035, 3027). We observed that, in some cases, DMI quantified turnover rates demonstrate alignment with the quantified turnover rates obtained without DMI in terms of the synchronization among heterocomplex interactors in the ISO and CTRL conditions (e.g., CPX-2055, 16).

We also observed DMI quantified turnover to provide insight into the change in complex synchronization between the ISO and CTRL conditions. We zoomed in to analyze a select number of these complexes where turnover exhibited incoherence in the ISO experimental group, yet suggested coherence in the CTRL group: (1) UBC13-UEV1A ubiquitin-conjugating enzyme E2 complex; (2) Mitochondrial NIAUFX iron–sulfur cluster (ISC) assembly complex; (3) AP-2 Adaptor complex, alpha1 variant; (4) Laminin-211 complex (Figure 4D). We further compared the change in coherence (one way ANOVA). The analysis indicated a decrease in coherence across all four complexes, suggesting that mismatches in turnover rates within complexes critical to cardiac function could play a role in the pathophysiology of heart failure: (1) The ubiquitin-conjugating enzyme complex plays a key role in the process of eliminating damaged and/or misfolded proteins in response to cardiac stress;^38^ (2) The Mitochondrial NIAUFX iron–sulfur cluster (ISC) assembly complex is required for the de novo synthesis of iron–sulfur (Fe–S) clusters within mitochondria. Defects in ISC biogenesis are associated with disorders of mitochondrial import, export, and translation and have been linked with cardiomyopathies;^39,40^ (3) AP2, a membrane-bound complex, interacts with clathrin in the plasma membrane to form clathrin-coated vesicles, controlling intracellular trafficking in endocytosis and playing a crucial role in autophagy and lysosomal protein degradation;^41^ (4) Laminin 211, an extracellular matrix protein, functions to stabilize the basement membrane and muscle fibers during cardiac contraction.^42^ This analysis underscores the utility of DMI in proteomics, providing preliminary insights into protein dynamics that merit further investigation.

### The DMI Pipeline Recovers Dynamics of Potential Biomarkers

**Figure 5:**
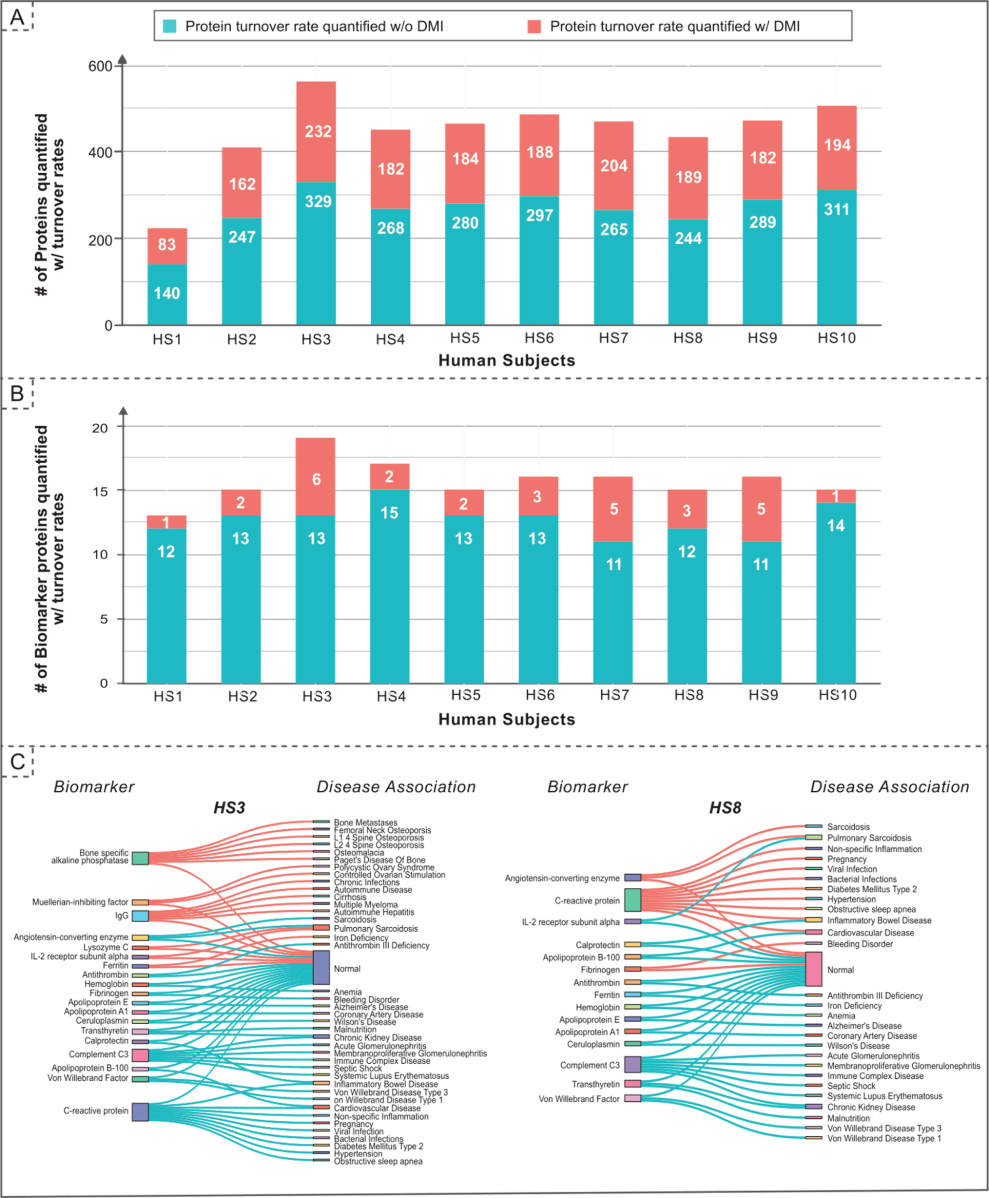
DMI pipeline enhances protein quantification in human samples. The bar chart presents a comparison of protein turnover rates quantified with and without Data Imputation (DMI) across 10 human subjects when examining both the plasma proteome (A) and the biomarkers it carries (B). The application of data imputation results in a significant increase in quantifiable protein turnover rates, with a 60–70% improvement observed in the proteome and a 10% enhancement noted in individual biomarkers. (C) We elucidate biomarker–disease associations in the data set gained with DMI, it reveals three patterns: (1) new disease associations (e.g., HS8: C-reactive protein → Hypertension); (2) new evidence supporting existing disease association (e.g., HS3: Lysozyme C and IL-2 receptor subunit alpha → Pulmonary Sarcoidosis); and (3) adding to the list of markers pre-DMI (e.g., HS3: Ferritin or HS8: Fibrinogen). Biomarker-disease associations across all human subjects are detailed in Figure S3 (please see Supporting Information).

To further demonstrate the capabilities and effectiveness of our Data Multiple Imputation (DMI) pipeline, we applied our workflow to a human plasma temporal proteomics data set.^29^ Similarly, DMI significantly enhanced the number of proteins that can be quantified in each subject by an additional ∼60% (Figure 5A). This substantial improvement in protein coverage allows for an improved understanding of the proteome dynamics landscape, thereby broadening the scope of potential clinical applications.

To illustrate a clinical application, we investigated whether additional DMI recovered biomarkers can be quantified. A list of biomarkers from MarkerDB^35^ was retrieved and compared with the protein list generated with and without the application of DMI. Our analysis revealed that DMI successfully recovered an additional 2–3 biomarkers per subject on top of the original ∼10 biomarkers (Figure 5B). To assess the potential of the additionally identifiable biomarkers to impact diagnostic and prognostic assessments, we obtained biomarker–disease associations curated from MarkerDB. We observed that certain biomarkers can be highly specific to particular diseases when outside their normal ranges. However, most biomarkers can be less specific and indicative of a family of diseases (e.g., C-reactive protein can be associated with any host of inflammation-related diseases, while human growth hormone can be linked to growth deficiency or acromegaly). Therefore, the ability of imputation to capture additional plasma biomarkers has high clinical utility. It can provide additional corroboration for a specific disease differential, confirm the absence of disease, or indicate the potential of other disease (Figure 5C and S3). This comprehensive biomarker profile helps strengthen the overall differential diagnosis and directs the clinician toward further clinical investigation.

Temporal cardiovascular proteome dynamics studies often suffer from missing data problems, and it hinders our ability to gain insights from these valuable data resources. In many cases, mechanisms contributing to missing values are complex and typically stem from a combination of Missing Completely at Random (MCAR), Missing at Random (MAR), and Missing Not At Random (MNAR).^22^ Therefore, methods that can accommodate various combinations of missing data patterns are necessary. The DMI method discussed herein is effective for handling MCAR and MAR data but can also accommodate MNAR patterns followed by some sensitivity analysis,^26^ thus addressing various types of missing data scenarios. However, it is advisable to select specific imputation methods tailored to the nature of the missing mechanism when such information is known or strongly assumed.

Our DMI pipeline allows users to adjust the parameters of imputation to meet the demands of their proteomics data analysis in the following aspects: it provides a default regression model but allows users to choose preferred regression methods in the multiple imputation process; allows users to specify the minimum samples required for imputation, which depends on the specific experimental design; allows selection of the number of data sets, *m*, for multiple imputation, which should be chosen based on the computational resources available and reliability desired.

As demonstrated in our study, a primary advantage of the DMI pipeline is to better address uncertainties in handling missing data compared with ad hoc or single imputation methods. We showed the benefit of our DMI pipeline for protein turnover rates inference by applying it to the cardiac temporal data sets.

## Conclusion

Missing values is a common issue in MS-based proteomics studies and especially in proteome dynamics data sets. Our DMI pipeline successfully addressed missing data challenges and demonstrated its utility on two distinct existing temporal proteomics data set. In brief, the DMI pipeline captured additional protein turnover rates. These recovered protein dynamics enable a more detailed view of biological pathways, protein complexes, and plasma biomarkers previously obscured, thereby enhancing our understanding of biological insights into the underlying protein dynamics in cardiovascular diseases. In summary, our DMI pipeline can expand the scope of proteome characterization in temporal data sets.

## Supporting Information Available

(1) We simulate a data set with missing values and evaluate the performance of imputation methods (DMI and DSI) on imputing the missing values, the result is presented in Figure S1; (2) we performed pathway enrichment on proteins quantified with and without imputation to determine all biological processes captured with DMI in the proteome, the result is presented in Figure S2; and (3) we investigated patterns of biomarker–disease associations for biomarkers contained in human plasma proteome with DMI, the result is presented in Figure S3 (PDF)
