# Supplementary material for: Missing Values in Longitudinal Proteome Dynamics Studies: Making a Case for Data Multiple Imputation
Source: J Proteome Res. 2024 Aug 27;23(9):4151–62. doi: 10.1021/acs.jproteome.4c00263 (PMC11385379; doi:10.1021/acs.jproteome.4c00263)
Supplement: Supplementary file 1 — pr4c00263_si_001.pdf [file pr4c00263_si_001.pdf]

Title: Missing Values in Longitudinal Proteome Dynamics Studies: Making a Case for Data Multiple Imputation

Authors and Affiliations:

Yu Yan[1,2,3#*], Baradwaj Simha Sankar[1,3#], Bilal Mirza[1,2#], Dominic C.M. Ng[1,2,3], Alexander R. Pelletier[2,4], Sarah D. Huang[1,2], Wei Wang[2,4], Karol Watson[1,3], Ding Wang[1,2,3], Peipei Ping[1,2,3,4*]

[1]Departments of Physiology and Medicine @ University of California, Los Angeles (UCLA) School of Medicine.

[2]NHLBI Integrated Cardiovascular Data Science Training Program @ UCLA.

[3]NIH BRIDGE2AI Center at UCLA & NHLBI Integrated Cardiovascular Data Science Training Program at UCLA, Suite 1-609, MRL Building, 675 Charles E. Young Dr. South, Los Angeles, CA 90095-1760, USA.

[4]Department of Computer Science and Scalable Analytics Institute at UCLA School of Engineering, CA 90095, USA.

# Equal contributions

* Correspondence

TABLE OF CONTENTS

We include “Supporting Information” on demonstrating the utility of DMI pipeline on two distinct datasets and their enhanced biomedical insights gained by using this pipeline.

1. **Figure S1**: DMI demonstrates better imputation performance in simulated datasets.
2. **Figure S2**: DMI enhances protein turnover rate detection in biological pathways.
3. **Figure S3**: DMI recovers dynamics of potential biomarkers.

SUPPORTING INFORMATION:

The following supporting information is available free of charge at ACS website http://pubs.acs.org.

We simulated datasets with missing values and evaluated the performance of imputation methods (DMI and DSI) on imputing the missing values, the result is presented in Figure S1.

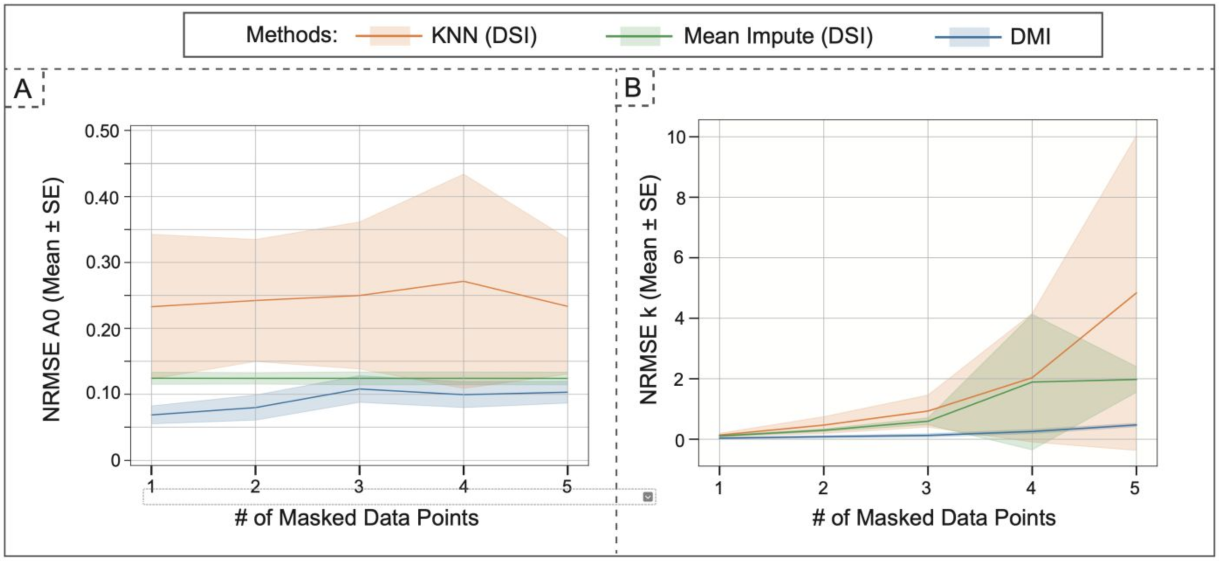

Figure S1. **DMI demonstrates better imputation performance in simulated datasets**. To simulate the different levels of missingness, we have created five masked scenarios in silico, where 1 time point and up to 5 time points out of the complete 7 time points were randomly masked. On each of these masked datasets (scenarios), we applied three imputation methods to recover the datasets: 1) DMI; 2) Single Imputation using Mean (DSI); and 3) Single Imputation using K-nearest neighbor (DSI). Each masked dataset undergone the DSI workflow produced one imputed dataset, resulting in a total of 5 imputed datasets. Each masked dataset that underwent the DMI workflow produced 10 imputed datasets for each of the masked configurations, resulting in a total of 50 imputed datasets. The imputed A0 values were compared against the ground-truth A0 values to evaluate the ability of imputation methods that can faithfully recover the original data. Subsequently, we conducted kinetic analysis to quantify the turnover rates on each masked dataset for each imputation method independently. The turnover rate estimated using imputed A0 time series were compared against the ones using completely observed A0 time series to evaluate the ability of imputation methods to capture the temporal dependencies among values. The accuracy of the imputation methods was quantified using the normalized root mean square error (NRMSE). As illustrated in the figure, DMI (blue) consistently demonstrates superior performance in recovering missing data, evidenced by the lower Normalized Root Mean Square Error (NRMSE) values and narrower Standard Error (SE) range in comparison to DSI methods in either single time point level (A0 in panel A) or time series level (turnover rate in panel B). In addition, DMI is more resilient to higher levels of missingness compared to DSI, as its NRMSE and SE are less affected with the increasing number of masked data points.

We performed pathway enrichment on proteins quantified w/wo imputation to determine all biological processes captured with DMI in the proteome, the result is presented in Figure S2.

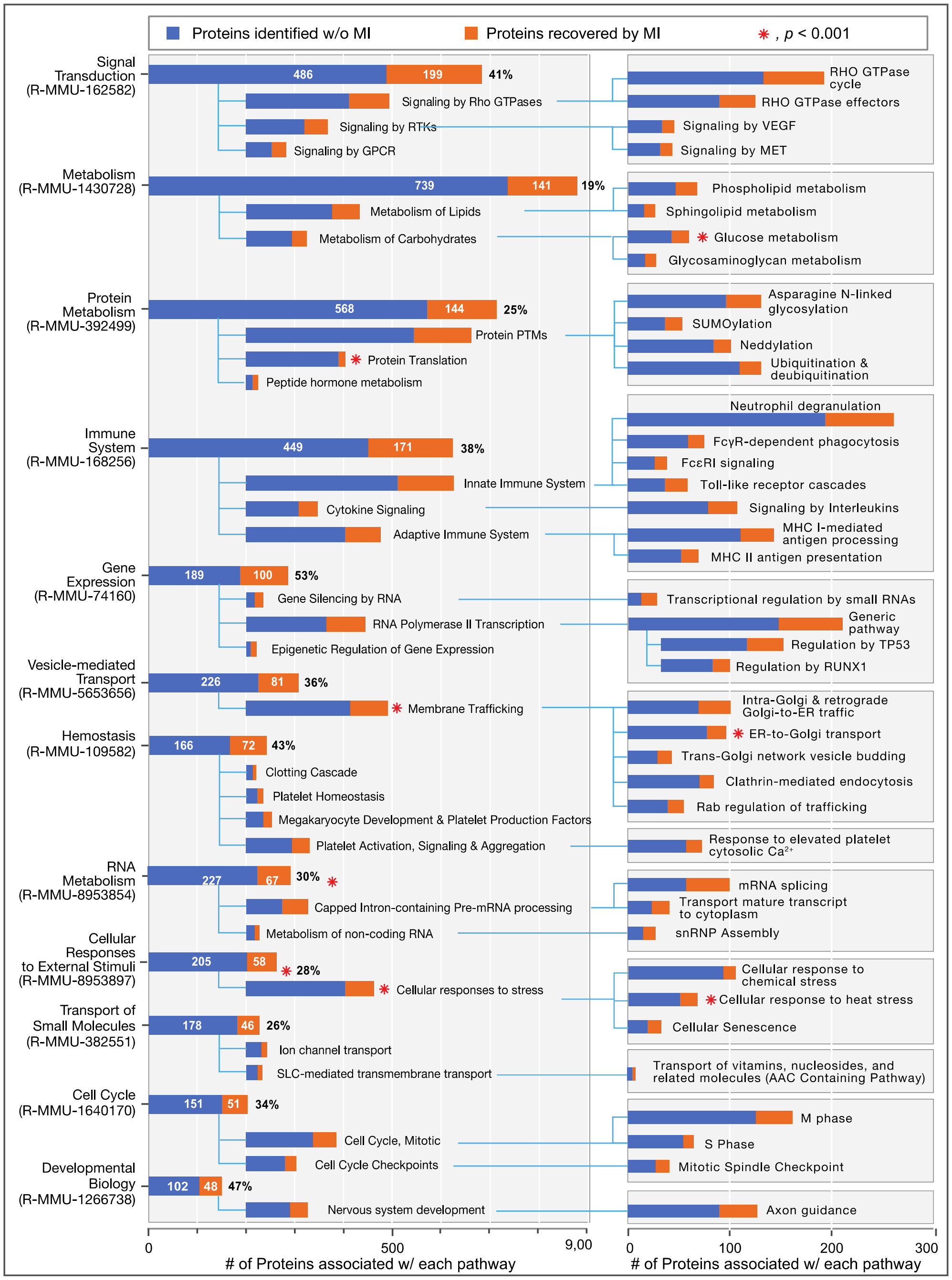

Figure S2. DMI enhances protein turnover rate detection in biological pathways. The DMI pipeline leads to enhanced protein turnover rate detection in multiple biological pathways, but it has limited impact on others. For example, in the "Protein Metabolism" pathway, DMI captures the turnover rates of additional 144 proteins (in orange) on top of the 568 proteins (in blue) without imputation. A "*" sign indicates a significant enrichment of the pathway with the recovered proteins based on pathway enrichment analysis.

We investigated patterns of biomarker-disease associations for biomarkers contained in human plasma proteome with DMI, the result is presented in Figure S3.

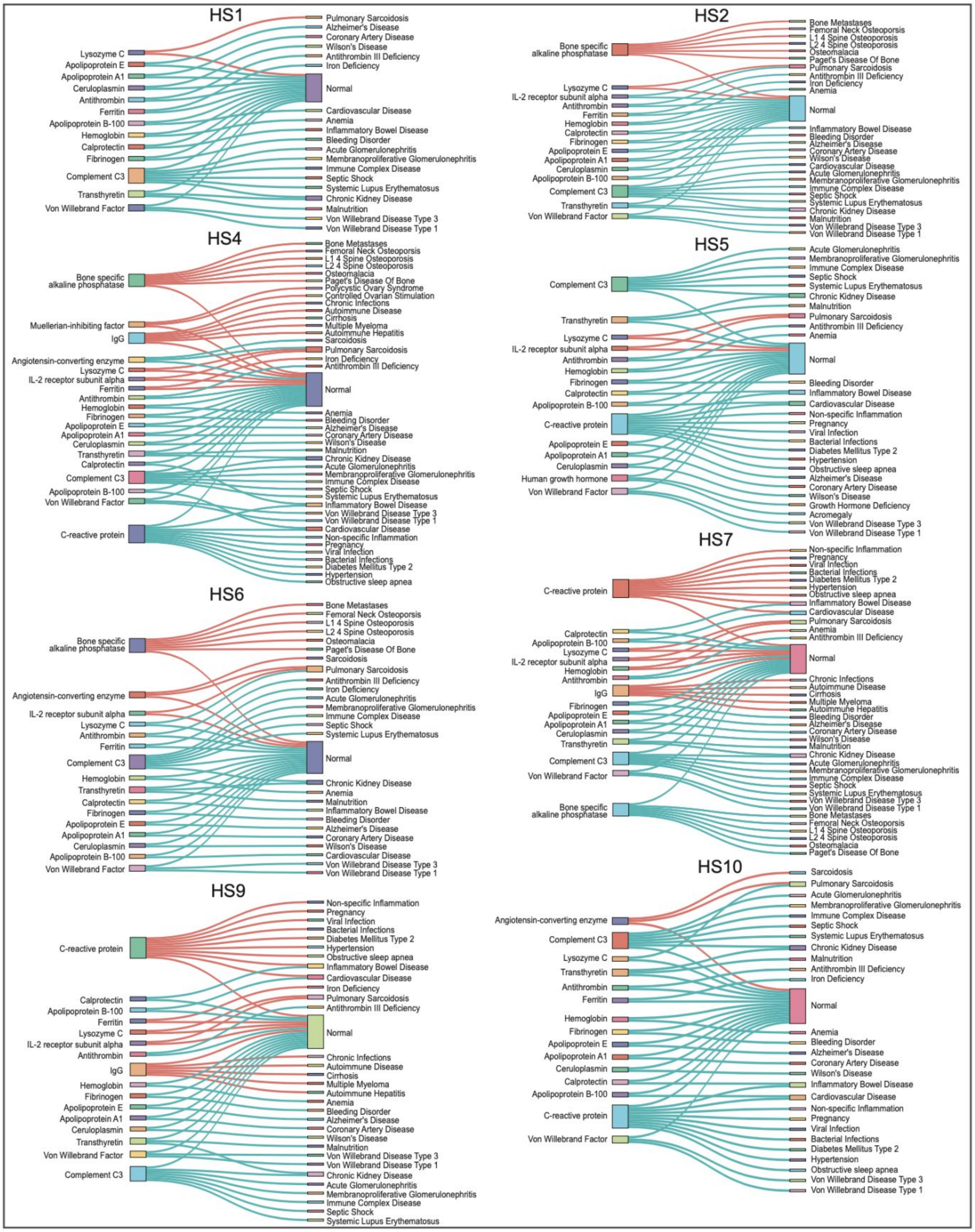

Figure S3. **DMI recovers dynamics of potential biomarkers.** Analysis of biomarker-disease associations across subjects reveals that DMI-imputed biomarkers either strengthen existing disease associations or provide evidence for new ones. A total of 30 additional biomarkers are discovered, 2 of which are new biomarkers only discovered with DMI across 10 subjects. This leads to a total of 116 additional biomarker-disease associations, 7 of which are new biomarker-disease associations that were not detected pre-imputation. Please see **Figure 5C** for examples from samples HS3 and HS8.
